# Annotations

**Published:** 1900-03-17

**Authors:** 


### Annotations.

### The Midwives' Bill.

The Midwives' Registration Bill had unexpected good luck in passing the second reading last Friday. It now goes before a Grand Committee, where its provisions will receive individual attention, and will probably be amended in several particulars. We can hardly doubt that the resolution passed by the Executive Committee of the General Medical Council on February 26th will receive the attention it deserves. This resolution drew attention to the fact that the Bill now before Parliament does not make provision, as advised by the Council, for requiring the licensed midwives to apply for medical assistance in every case which presents difficulty, danger, or disease on the part of the mother or the new-born child. In view of the extreme differences of opinion which exist as to the proposed scope and aim of the Bill, and as to the sort of training which midwives ought to be compelled to undergo, it would also be well that some guidance on this subject should be incorporated in the body of the Bill, instead of all these details, which are really matters of principle, being left to the decision of such an ever-varying body as the Central Midwives' Board. Perhaps a clause might also be introduced concerning the quack man-midwife. At present he is not existent in any large degree, but if it should so happen that the Midwives' Board should screw up the qualification of the midwives, and should thus make midwifery a remunerative business confined to a close corporation, the quack man-midwife would soon come to the fore, for as the Bill stands now the midwife whom it controls is by the terms of the Bill "a woman."

### The Birmingham Consultative Institute.

It appears from the announcement made in *Forward*, the organ of the Birmingham Hospital Saturday Fund, that a consulting physician has been appointed to the above institute. Not only so, but all the machinery for advertising for patients seems to be in an advanced stage. It is announced that first-class consulting-rooms have been taken, and that bills, pamphlets, and all detailed information will be supplied to the manufactories, trading establishments, and mercantile offices of the city. If this is to be taken as representing the true state of affairs we need hardly say how absolutely opposed to the best traditions of the medical profession is the whole undertaking. We can, indeed, with difficulty bring ourselves to believe that any man of standing could accept such an appointment if he recognised that his introduction to practice would be of so degrading a nature as is indicated above. No one objects to medical men charging exactly what they like for their services. There are some very good fellows, quite within the pale of professional brotherhood, who, we believe, give their advice for a shilling. Let no one think, then, that it is because the advice to be given at the Birmingham Consultative Institute is to be cheap that we object to it. Nothing of the kind. In fact, we do not think it is cheap. There are plenty of highly-qualified medical men in Birmingham who would give quite as good advice for five shillings as is at all likely to be supplied for the Consultative Institute half-guinea. What is objectionable is, on the one hand, the system according to which the services of the "consultants" are to be exploited by the managers; and, on the other hand, the advertising by which they are to be introduced to practice. According to *Forward*, the Consultative Institute has appointed as its consulting physician a young gentleman who, so far as the information in the "Medical Directory" tells us, became qualified in the year 1895, and obtained his M.D. degree in 1897, and has not yet held any hospital appointments. Far be it from us to say a word against this gentleman as a practitioner, but is he a consultant? Is he, in the words of *Forward*, one who, "by reason of his special training, experience, and research," would be called in to give "the best possible consideration and advice" in regard to cases in which "ordinary practitioners" find "better advice and more advanced treatment" necessary; cases which are "beyond their scope or power of diagnosis"; cases in which the patient is advised to take advantage of the "higher skill and knowledge" of some consulting physician? It is saying nothing against the gentleman selected to point out that it is beyond the power of any body of men, never to mention a consultative institute, to confer such a position on a practitioner of five years' standing. The thing is too absurd for words.

### A Lesson of the Winter.

In a report to the Corporation, Dr. Sedgwick Saunders, medical officer of health to the City of London, has drawn attention to the cruel and injurious practice of using salt to facilitate the removal of snow from the streets. He points out that the immediate effect of the addition of salt to snow is to reduce its temperature from 32 deg. to zero Fahrenheit, and says that the suffering to bare-footed or ill-shod persons and the injury caused by the chilling effect which the mixture has upon the tender frog of the horses' feet, have been so often explained that he is surprised that such a cruel and barbarous practice should be continued. We can fully bear out all that Dr. Saunders says in regard to the evil effects of salted snow upon the boots and persons of those whose misfortune it is to have to paddle through it. Not merely does it at the moment produce an icy coldness, the intensity of which is infinitely greater than any that is produced by walking in ordinary snow, but, unfortunately, boots once so pickled are rendered unfit for use for a long time to come. It is quite unnecessary to expatiate upon the evils of cold feet, and when boots are salted it is impossible to keep them either warm or dry. Directly they touch snow they become saturated with a freezing mixture. We do not deny that there are occasions when the use of a little salt may be useful and, perhaps, almost necessary, for the rapid removal of ice when this is frozen hard and solid on the pavement. As a means for facilitating the scraping and sweeping by which such ice is being removed the use of salt may now and then be justifiable, but never for the removal of snow; and even when used for ice the resulting mixture should never be left on the ground. The cost to the community in spoilt health, not to mention spoilt boots, is far greater than the apparent saving effected by salting snow.

